# Self-Assembly of Polymers and Their Applications in the Fields of Biomedicine and Materials

**DOI:** 10.3390/polym16152097

**Published:** 2024-07-23

**Authors:** Lina Hu, Shujing Zhou, Xiumei Zhang, Chengyang Shi, Yifan Zhang, Xiaoyi Chen

**Affiliations:** School of Pharmacy, Jiamusi University, Jiamusi 154007, China; hulina19870412@126.com (L.H.); zhshj2003@163.com (S.Z.); yingminmei@163.com (X.Z.); shichengyang@163.com (C.S.); 13796098193@139.com (Y.Z.)

**Keywords:** self-assembly, carbohydrates, proteins, synthetic polymers, materials

## Abstract

Polymer self-assembly can prepare various shapes and sizes of pores, making it widely used. The complexity and diversity of biomolecules make them a unique class of building blocks for precise assembly. They are particularly suitable for the new generation of biomaterials integrated with life systems as they possess inherent characteristics such as accurate identification, self-organization, and adaptability. Therefore, many excellent methods developed have led to various practical results. At the same time, the development of advanced science and technology has also expanded the application scope of self-assembly of synthetic polymers. By utilizing this technology, materials with unique shapes and properties can be prepared and applied in the field of tissue engineering. Nanomaterials with transparent and conductive properties can be prepared and applied in fields such as electronic displays and smart glass. Multi-dimensional, controllable, and multi-level self-assembly between nanostructures has been achieved through quantitative control of polymer dosage and combination, chemical modification, and composite methods. Here, we list the classic applications of natural- and artificially synthesized polymer self-assembly in the fields of biomedicine and materials, introduce the cutting-edge technologies involved in these applications, and discuss in-depth the advantages, disadvantages, and future development directions of each type of polymer self-assembly.

## 1. Introduction

### 1.1. Brief Introduction to Polymers and Self-Assembly

Polymers, also known as high-molecular-weight polymers, generally refer to compounds with a relative molecular weight of several thousand to several million. The vast majority of high-molecular-weight compounds are mixtures of many homologous compounds with different relative molecular weights. Therefore, the relative molecular weight of high-molecular-weight compounds is the average relative molecular weight. The relative molecular weight of polymer compounds is one of the most fundamental physical and chemical properties, which is of great significance for studying their structure and properties. The commonly used measurement methods include end-group analysis, the boiling-point rise method, freezing-point drop method, vapor pressure drop method, osmotic pressure method, light scattering method, and the ultracentrifugation sedimentation method. Among them, end-group analysis is the most commonly used method, which determines the relative molecular weight of polymer compounds by measuring the content of end functional groups in them. This method is easy to operate, highly accurate, and suitable for various types of polymer compounds. The boiling-point rise method calculates the molecular weight of solutes by measuring the change in boiling point of a solution. This method is applicable to non-volatile substances such as polymers. However, since other factors in the solution may also affect the boiling point, it is necessary to calibrate the freezing-point drop during the experiment. The law of freezing-point drop is to calculate the molecular weight of solutes by measuring the freezing-point drop of the solution. This method is applicable to water-soluble polymers but it also requires calibration of the experimental results. The vapor pressure drop method calculates the molecular weight of the bath by measuring the vapor pressure of the bath. This method is applicable to volatile substances such as oligomers and monomers. However, due to other factors in the solution that may also affect vapor pressure, calibration is required during the experimental process. The osmotic pressure method calculates the molecular weight of solutes by measuring the osmotic pressure of a solution. This method is applicable to water-soluble polymers but it also requires calibration of the experimental results. Polymer compounds are composed of thousands of atoms connected by covalent bonds. Although their relative molecular weight is large, they are all connected in simple structural units and repeated ways. According to the different sources of polymers, they are further divided into natural polymers and artificially synthesized polymers [1,2]. Natural polymer materials have good biodegradability and can reduce environmental pollution. Most synthetic materials are artificially synthesized polymer materials that are not easily degraded by nature, thus causing adverse effects on the environment. The emergence of functional polymers has advanced the application of synthetic polymers to a more refined and advanced level. It not only promotes industrial and agricultural production and cutting-edge technology but also plays an important role in exploring the mysteries of life, conquering cancer, and treating genetic diseases. It is estimated that the population on Earth will exceed 10 billion in the near future, and food, energy, environment, resources, and other issues will become more troublesome for human society. Polymer science will play an important role in this regard. The application of fat substitutes developed from natural polymers such as proteins, fats, or carbohydrates not only helps reduce the calorie and fat content of food but may also provide additional health benefits such as lowering cholesterol. With the continuous development of materials science and electrochemical technology, the application prospects of polymer materials in fields such as solar cells, lithium–ion batteries, and supercapacitors will become more extensive and broader. In addition, biodegradable materials such as starch plastics and photo-degradable materials can be developed using polymers as raw materials.

Carbohydrates are one of the most abundant and important biomolecules in nature. Besides compounds related to energy, carbohydrates can be roughly divided into two categories: carbohydrates as substances and carbohydrates as information. As substances, carbohydrates are abundant in the extracellular matrix of animals and in the cell walls of various plants, bacteria, fungi, etc., serving as scaffolds. Some common polysaccharides are biocompatible materials with controllable rigidity and functionality, forming polymer biomaterials widely used in drug delivery, tissue engineering, and other fields [3]. As information, carbohydrates typically refer to glycans in glycoproteins, glycolipids, and proteoglycans that bind to proteins or other carbohydrates, thereby affecting cell–cell and cell–matrix interactions. These polysaccharides can be simplified into synthesized sugar polymers, glycolipids, and glycoproteins, which can be provided through polymerization, multi-step synthesis, or semi-synthetic strategies. The informational effects of carbohydrates can not only serve as targeting reagents but also as immune antigens and adjuvants [4].

Proteins are the primary component of constructing complex nanostructures in nature, which can perform complex tasks and chemical transformations. Approximately 70–80% of proteins are considered to be permanently oligomeric. That is to say, they are composed of multiple proteins that bind together in precise spatial organization through non-covalent interactions. Mastering protein–protein interactions (PPIs) will also enable people to obtain novel biomaterials with the most beloved and versatile building blocks in nature [5].

Self-assembly refers to a technique in which basic structural units (molecules, nanomaterials, micrometers, or larger-scale substances) spontaneously form ordered structures. During the self-assembly process, the basic structural units spontaneously organize or aggregate into a stable structure with a certain regular geometric appearance based on non-covalent interactions. Self-assembly technology is a method of spontaneously assembling molecules, atoms, or nanoparticles into specific structures or functions. Its research progress covers new material development, understanding of self-assembly mechanisms, engineering control methods, and multi-scale self-assembly. In terms of application, self-assembly technology has been widely applied in fields such as nanoelectronic devices, nanomaterial preparation, biomedicine, etc., providing new solutions for electronics, sensors, drug delivery, etc. In the future, self-assembly technology is expected to achieve more complex and multifunctional self-assembly structures, including multifunctional self-assembly, self-healing materials, biomimetics, and quantum self-assembly, which will further promote technological innovation and industrial development [6,7,8,9,10].

### 1.2. Introduction to Polymer Self-Assembly

The principle of polymer self-assembly refers to the existence of some mutually attractive forces in polymer molecules, such as van der Waals forces, electrostatic interactions, hydrophobic interactions, etc. For example, the characteristic of ion-complementary peptides is the alternating arrangement of negatively charged amino-acid residues and positively charged amino-acid residues, which initiate molecular self-assembly through electrostatic interactions, hydrogen bonding, and van der Waals forces. Its hydrophilic and hydrophobic regions alternate and are divided into two ordered regions. Hydrophobic amino-acid residues fold to shield water molecules, while the hydrophilic region has regular and ordered positive and negative charges that attract each other. The formation of intermolecular hydrogen bonds accelerates the self-assembly of peptides, and the interlocking of ionic bonds increases the strength of the self-assembled structure. When these forces reach a certain level, molecules will spontaneously assemble into a certain structure. The self-assembly structure of objects includes spherical particles, nanowires, nanoplates, nanospheres, nano micelles, etc. The self-assembly process of polymers is achieved by adjusting the interactions between polymer molecules to achieve the desired assembly structure. Common processes include the solution method, thermal method, chemical deposition method, etc. The conditions required for polymer self-assembly include the following: polymer concentration in solution, temperature, pH value, ion strength, etc. Polymer self-assembled materials have many potential applications, including but not limited to the following aspects. Biomedical applications: Polymer self-assembled materials can be used in fields such as drug delivery, cell imaging, and tissue engineering. By designing and controlling polymer systems, nanocarriers with specific functions can be prepared for drug encapsulation and release. The special properties of the material also make it an ideal material for biological imaging and tissue repair. When designing polymer systems for drug encapsulation and release in biomedical applications, factors such as drug solubility, compatibility between drugs and carrier materials, and the drug carrier ratio need to be considered. Energy material applications: Polymer self-assembled materials can be used in fields such as solar cells, energy storage materials, and catalysts. Through the self-assembly of nanoparticles and the directing effect of polymers, nanomaterials with excellent optoelectronic properties and catalytic activity can be prepared. Sensor applications: Polymer self-assembled materials are also widely used in the field of sensors. By regulating the self-assembly of nanoparticles and the structure of polymers, highly sensitive and selective sensor materials can be prepared for detecting environmental changes and biomolecules. Photonics applications: Polymer self-assembled materials have important applications in the field of photonics. Through the self-assembly of nanoparticles and the directing effect of polymers, nanomaterials with special optical properties can be prepared for the preparation of photonics devices and optical information storage [11,12,13,14,15].

The self-assembly method of natural polymers mainly relies on non-covalent forces between molecules, such as hydrogen bonding, hydrophilic/hydrophobic interactions, electrostatic interactions, π-π stacking, etc. These forces promote peptide self-assembly spontaneously or are triggered by specific conditions and further form morphologically specific structures. This self-assembly mechanism provides an effective platform for biomedical applications such as drug delivery, gene therapy, and immunotherapy. The self-assembly method for synthesizing polymers involves the ordered arrangement of molecules to form particles, which has the characteristics of simple preparation and convenient operation but has poor stability and is prone to aggregation. The common self-assembly methods include the micellar method, reverse microemulsion method, and polymer solution deposition method. The polymer nanoparticles prepared by these methods have shown broad application prospects in biomedical applications, such as drug sustained- and controlled-release, gene transfer, etc. [16,17].

Here, we listed the self-assembly of natural polymers, including carbohydrates, proteins, and peptides as well as the self-assembly of synthetic polymers. Starting from their representative applications in the fields of biomedicine and materials, we briefly introduce their self-assembly methods and technologies and discuss the advantages and disadvantages of these technologies, as well as the future development directions of polymer self-assembly [18].

## 2. Self-Assembly and Application of Natural Polymers

### 2.1. Carbohydrate Self-Assembly and Application

Carbohydrates are naturally occurring compounds with biocompatibility and biodegradability, and provide excellent opportunities for chemical modification. In addition, they play an important role in many biological processes, including immune response, growth regulation, cell signaling, adhesion, and fertilization. By introducing specific biosensing or labeling groups on carbohydrates, reliable and specific interactions can occur with target molecules, achieving high sensitivity and selectivity for related analysis or detection. The functional design of carbohydrates involves a large amount of molecular structure design and evaluation work, which requires the use of advanced technologies such as computational simulation and high-throughput screening to achieve efficient and rapid design processes. Therefore, it is necessary to strengthen the research methods that combine calculation and experimentation to improve the accuracy and feasibility of carbohydrate functional design. Due to these characteristics, carbohydrates have been successfully integrated into amphiphilic structures and used in biomedical applications such as drug delivery, gene therapy, diagnostic imaging, and photosensitizer delivery [19].

**Figure 1:**
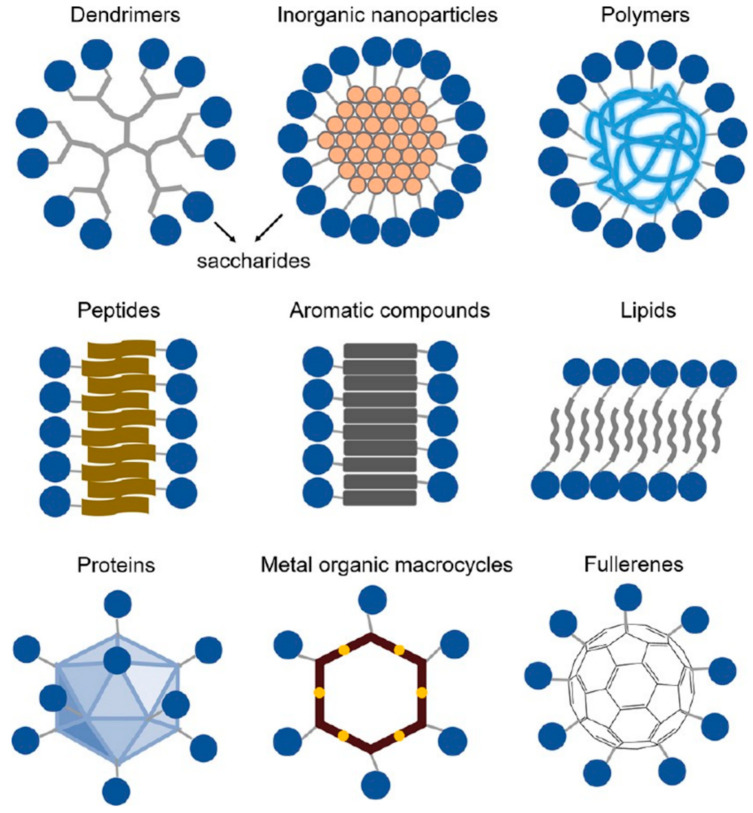
Precise illustration of multivalent carbohydrates with other compounds (e.g., dendrimers, polymers, peptides, proteins, etc.) as scaffolds [20]. Copyright 2022. Reproduced with permission from ACS.

Large-molecule carbohydrates contain sugars, cellulose, starch, glycoproteins, glycolipids, proteoglycans, etc. They are the most abundant and widely distributed organic compounds in nature. Their natural functions are related to energy metabolism, cellular structure, and molecular interactions. Their excellent characteristics, including abundance, biocompatibility, and biodegradability, highlight their indispensable role as building blocks in designing next-generation biomaterials [3]. In order to prepare carbohydrate-related biomaterials with uniform molecular weight, controllable molecular position, and functions to meet the needs of modern medicine and advanced biomaterials, other compounds such as polymers, dendrimers, aromatic compounds, lipids, peptides, and proteins are usually used as scaffolds to synthesize carbohydrate components (Figure 1). These modified compounds, with carbohydrates as functional groups, not only successfully mimic the natural functions of polysaccharides but also demonstrate potential applications in molecular recognition, antigenicity, anticancer therapy, and immunotherapy [20,21,22,23].

#### 2.1.1. Application of Carbohydrate Self-Assembly in the Field of Biomedicine

##### Identification Function

One important function of natural carbohydrates—such as glycolipids and glycoproteins—is their interaction with other proteins or sugars, which can mediate information transmission in cells and cellular environments and regulate life activities including cell differentiation, proliferation, and migration [24]. In the past 20 or even 30 years, this research field has attracted great attention, shifting from relatively direct multivalent binding enhancement to intrinsic cellular functions triggered by molecular recognition of carbohydrates and lectins. The precise carbohydrate assembly using various molecules as scaffolds can provide a surface with multivalent presentation to enhance this molecular interaction.

Wang prepared uniform nanoribbons using a self-assembled glycopeptide conjugated polymer (GPC) system, which were attached with multivalent homogeneous oligosaccharides. The system successfully transformed the structural differences of oligomannose into different binding affinities to C-type lectin receptors (CLRs). These findings highlight the potential of GPCs as vaccine adjuvants and demonstrate their multifunctionality in utilizing the biological functions of carbohydrates [25].

Banger synthesized amphiphilic-functionalized polysaccharides through solid-phase polymer synthesis and applied them in self-assembled micelles and large monolayer vesicles as a simplified model of cellular glycocalyx. In addition, the aggregation-induced luminescent material was introduced into amphiphilic sugar macromolecules, and fluorescence was not displayed when the molecules were free in solution. To obtain micelles and vesicles that do not display fluorescence or only display very small fluorescence, sugar macromolecules carrying bound polysaccharide moieties, luminescent bodies with sugar macromolecules, or other amphiphilic compounds in self-assembled structures that do not have binding moieties or luminescent bodies can be combined. An increase in fluorescence can only be observed when the binding polysaccharide motif aggregates through interaction with multivalent lectin receptors. Therefore, it was possible to detect and locate aggregation events within these self-assembled structures [26].

Bi reported a one-dimensional sugar nanorod (GNR) that exhibited superior targeted sensing and killing effects on Gram-negative *Escherichia coli* (*E. coli*) through the self-assembly of a clear β-cyclodextrin-based glycoconjugate (RMAn), mainly through multivalent recognition between mannose on the nanorod and lectin on the surface of *E. coli*. There are very few targets that only cause killing to fungi without damaging human cells. The prospect of using the trehalose biosynthesis pathway as a target for antifungal drugs is very promising but further research is still needed on the functions of some key proteins in this pathway. Due to the distribution of this pathway in many organisms, its inhibitors may affect other human symbionts, which requires further research. Another noteworthy issue in this field is the efficacy of specific drugs in specific infectious microenvironments. Although the data mentioned in this article suggest that trehalose biosynthesis may play a role in established infections, this still needs to be fully validated through experiments. In addition, the combination of trehalose metabolism inhibitors with existing cell-wall-targeted drugs may become an efficient combination therapy. This work described a targeted and effective sensing and antibacterial platform based on glycoconjugates, which has potential applications in the treatment of infections caused by pathogenic microorganisms [27].

##### Anticancer Treatment and Immunity

As we mentioned, carbohydrates on the cell surface are crucial for information exchange between cells, especially during tumor progression and immune response processes. A hallmark feature of cancer is the abnormal synthesis of incomplete O-glycans, present in mucins and other glycoproteins, represented by Tn and T antigens and their sialylated glycoproteins, sialyl Tn (STn) and sialyl T (ST). Secreted glycoproteins expressing STn often appear in the blood of cancer patients. Due to their rarity in normal tissues, these polysaccharides can serve as prognostic biomarkers and therapeutic targets. Shortened O-glycans cause immune responses in patients, therefore, vaccines targeting these polysaccharides have been developed. As an alternative, incomplete O-glycans can serve as targets for various therapeutic antibodies and chimeric antigen receptor (CAR) T-cell therapies. During the process of tumor development, tumor-related glycosylation typically changes by increasing the branching of complex N-glycans, truncating O-glycans and altering sialylation. It inhibited the anti-cancer immune response by binding to sialylic acid-binding immunoglobulin-type lectins (Siglecs) expressed by immune cells including natural killer cells (NKs), dendritic cells (DCs), macrophages, etc. [28,29,30].

Brito reported that aromatic N-glucosides selectively inhibited cancer metabolism through two coexisting mechanisms: by depriving glucose uptake through competitive binding in GLUT1 glucose binding, and by forming a chelated nanoscale supramolecular network through local (biocatalytic) self-assembly on the cell surface [31].

Functionalized shells of various poly (dl lactide)-b-poly (acrylic acid) (PDLLA-b-PAA) spherical micelles and poly (l-lactide b poly (acrylic acid) (PLLA-b-PAA) cylindrical micelles with mannose were used to prepare sugar nanoparticles (GNP) of different shapes and sizes. All of these GNPs exhibited good biocompatibility (up to 1 mg/mL), which can more effectively induce inflammatory responses, especially interleukin-6 [32].

Chemists have designed a carbohydrate-type molecule that can self-assemble into a nanofiber network to surround and kill bone cancer cells. Meanwhile, since the self-assembly process is triggered by an enzyme that overexpresses cancer cells, this molecule has no effect on healthy cells [33].

A carefully designed oligomeric azobenzene-grafted mannose was synthesized using a step-by-step iterative method and “click” chemistry. The obtained oligomers had precise structures and exhibited multifunctional assembly forms and chirality that were responsive to light. These different assembly forms exhibited different abilities in inhibiting cancer cell proliferation and stimulating dendritic cell maturation [34].

Self-assembling glycopeptide complexes can be used to generate a series of multivalent glycosylated nanoparticles, which may interact with lectins targeting natural carbohydrates. This composite bio-ink had a low water absorption capacity (2.5%) and degraded within 3 weeks in the presence of lysozyme. The mechanical properties showed an elastic modulus of approximately 15.5 kPa. Rheological analysis showed that the bio-ink sample has a higher storage modulus at 37 °C. After 14 days of scaffold culture in osteogenic medium, the ALP activity at 36.8 units/mL showed high cellular activity. These results indicated that the incorporation of osteogenic nano hydroxyapatite and nanofiber aggregates enhanced the overall osteogenic and physicochemical potential of thermosensitive bio-inks. Through in vitro and in vivo immune experiments, it has been demonstrated that this glycosylated nanoparticle can trigger an immune response in receptors, thus acting as an immune activator. This work provided important reference for the development of structurally controllable carbohydrates based on self-assembled glycopeptide conjugates [35].

##### Tissue Engineering

Chitosan-based thermosensitive bio-ink can be a potential choice for bone tissue engineering due to its excellent biocompatibility and cross-linkless gel at physiological temperature; however, their low mechanical strength, poor printability, and low post-printing cell viability are some of the limitations. In this work, self-assembled nanofiber aggregates of chitosan and gelatin were prepared and incorporated into chitosan-based bio-inks to enhance printability, mechanical properties, post-printing cell viability, and proliferation. The results indicated that the incorporation of osteogenic nano hydroxyapatite and nanofiber aggregates enhanced the overall osteogenic and physicochemical potential of thermosensitive bio-ink [36].

Two kinds of synthetic supramolecular hydrogels formed by biurea amphiphiles containing bioactive ligands of lactose bionic acid (LBA) and maltose bionic acid (MBA) were used as in vitro cell culture matrices. Their fibrillar and dynamic properties simulated the basic characteristics of the extracellular matrix (ECM). Carbohydrate amphiphiles self-assemble supramolecular fibers in water, and hydrogels were formed by bundles through the physical entanglement of fibers. The gel of the two amphoteric affinities showed good self-healing behavior but their hardness was significantly different. They exhibited excellent biological activity in liver cell culture. Both carbohydrate ligands used were believed to bind to the asialoglycoprotein receptors (ASGPRs) in hepatocytes, thereby inducing spheroid formation when HepG2 cells were seeded on two supramolecular hydrogels. Ligand properties, ligand density, and hydrogel hardness affected cell migration and the size and number of spheres. Two synthetic supramolecular hydrogels, formed from bis-urea amphiphiles containing lactobionic acid (LBA) and maltobionic acid (MBA) bioactive ligands, were applied as cell culture matrices in vitro. Altogether, these results showed that both LBA and MBA gels promote the formation of the spheroids but their differences in concentration, related stiffness, and ligand density may result in variations in spheroid diameters and formation efficiency. The results also indicated that the self-assembled carbohydrate-functionalized hydrogel had potential as a matrix for liver tissue engineering [37].

##### Antimicrobial Agents

Tight binding between receptors on pathogens and the carbohydrates on host cells is crucial for the initial stage of pathogen invasion into host cells. Therefore, multivalent adhesives can be used to interfere with pathogen adhesion for the treatment of pathogens and prevent infection. Their in vivo activity remains to be tested and it is possible that such constructs, if used in this context, could elicit humoral responses that might potentially neutralize some of the interactions studied here. The high activity and fascinating (quasi)symmetric, high-surface-area morphology of these new glycoconjugates provide promising candidates for the development of both new antiviral agents as well as probes of larger-scale biological events. In the past few decades, various glycosylated materials have been proposed, among which glycosylated virus-like particles with self-assembly processes have shown better effects in virus inhibition because they are similar in size to pathogens and have the ability to accurately display a large number of glycosidic ligands [38,39,40,41].

Davis et al. reported a multivalent mannose VLP with a diameter of 32 nm, which can mimic natural pathogens in size. With a highly glycosylated surface and multivalent effect of glycosidic clusters, these mannoside VLPs can effectively block dendritic cell-specific intercellular adhesion molecule-3-grabbing non-integrins (DC-SIGN) in vitro, further preventing human dendritic cells and T lymphocytes from being infected by the Ebola pseudovirus [42].

Hack enberger and colleagues further reported that the spatially defined arrangement of sialic acid on bacteriophage capsids can effectively inhibit viral infection in vitro and in vivo through a good match between homologous trimeric hemagglutinin (HA) and the geometric shape of influenza A virus. This indicated that the development of new antiviral drugs had enormous potential. In order to treat bacterial infections, sugar-containing compounds have been used in clinical practice as a class of small-molecule antibiotics for decades [43].

**Figure 2:**
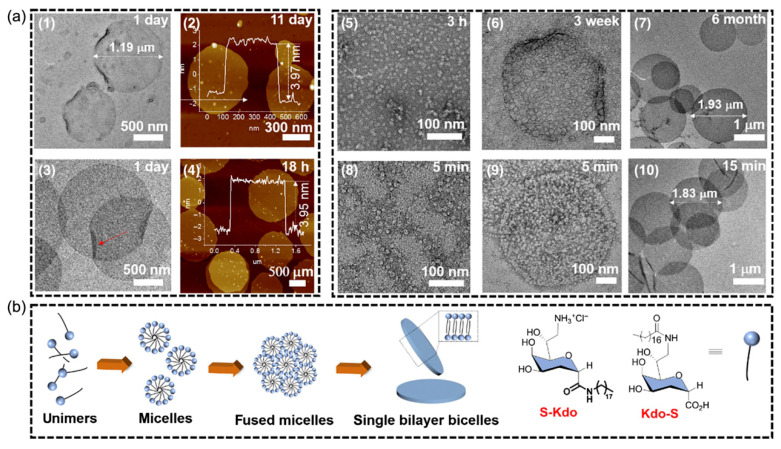
(**a**) Self-assembly of S-Kdo and Kdo-S. (1) TEM and (2) AFM images of bilayer structures formed by S-Kdo. (3) TEM and (4) AFM images of bilayer structure formed by Kdo-S. (5–7) TEM images of bilayer structure formed byS-Kdo at different time points. (8–10) TEM images of bilayer structures formed by Kdo-S at different time points. (**b**) Diagram of the formation process of the bicelles by S-Kdo and Kdo-S [44]. Copyright 2022. Reproduced with permission from CCS.

Currently, with the emergence of drug-resistant bacteria, carbohydrate assemblies have been proposed as a new antibacterial candidate to eliminate bacterial biofilms that may lead to chronic infections and antibiotic resistance. For example, Feng and his colleagues also observed the antibacterial activity of glycolipids by using Kdo (3-deoxy-Dmannose-2-octuronic acid) connected to lipid chains as a biomimetic bacterial membrane (Figure 2). By connecting lipids to the two different sides of Kdo, two glycolipid components, namely bicellular and band, were formed. In addition, the antibacterial test results showed that the self-assembled Kdo exhibited high antibacterial efficiency against Gram-positive strains (*Staphylococcus aureus* and *Escherichia coli*) rather than Gram-negative strains (*Escherichia coli* and *Pseudomonas aeruginosa*) [44].

#### 2.1.2. The Application of Carbohydrate Self-Assembly in the Field of Materials

With the advancement of purification technology, naturally occurring carbohydrates can be further chemically modified to produce sustainable biomaterials, some of which, such as hyaluronic acid, heparin, and chitosan, have been used in clinical and industrial settings due to their inherent biological activity. Bio-based materials have lower mechanical properties than petroleum-based materials but are easily degradable and belong to environmentally friendly materials; however, due to the complexity and diversity of their structure, the design and preparation of precise carbohydrate components for biomaterials are crucial compared to other design materials.

A series of all bio-based block copolymers (BCPs) with ABA-, A_2_BA_2_-, A_3_BA_3_-, and A (BA)_2_- as well as A_2_(BA)_2_- and A_2_(BA)_2_-type structures were synthesized, consisting of maltooligosaccharides (maltose, maltotriose, maltotriose, and malthexose; A-block) and poly (δ-decalactone) (PDL; B-block), to demonstrate the potential of oligosaccharides as novel bio-based elastomer hard-chain segments. These results indicated that oligosaccharides were a sustainable alternative to petroleum-derived synthetic hard-chain segments (such as polystyrene), opening up a new avenue for the design of all-bio-based soft materials [45].

In recent years, renewable cellulose-based ion-exchange membranes have become promising candidates for capturing green and rich osmotic energy; however, low power density and structural/performance instability pose a challenge for this cellulose membrane. Shi developed cellulose molecular self-assembly engineering (CMA) to construct environmentally friendly, durable, and scalable cellulose membranes (CMA membranes). The self-assembly engineering of cellulose molecules has constructed high-performance ion-selective membranes with the advantages of environmental protection, scalability, high wet strength, and stability, guiding sustainable nanofluid applications beyond blue energy [46].

Hydroxypropyl cellulose (HPC) is a sustainable cellulose derivative widely used in various fields due to its excellent biocompatibility and solubility. A new high-substitution HPC synthesis strategy was demonstrated, which was more effective than traditional methods. The highly substituted HPC prepared had significant thermal stability, excellent hydrophilicity, and satisfactory solubility. A unique self-assembly behavior emerged in solvent-free environments, characterized by the structural color and responsiveness to force [47].

Highly blocked CNSs (cellulose nanocrystal surfactants) were prepared using EPD (N′-ethylpropane-1,3-diamine) and TOCNC (TEMPO-oxidized cellulose nanocrystals), demonstrating a CO_2_-enhanced assembly method that generated a structured film at the oil–water interface to counteract capillary forces and control local displacement modes. This self-assembly strategy was of great significance in environmentally friendly and cost-effective applications, such as improving oil recovery, carbon dioxide geological storage, and water infiltration [48].

The self-assembly of carbohydrates and their derivatives through self-assembly has attracted long-term interest due to its potential applications in various fields such as biomedical and nanotechnology. Despite increasing efforts in carbohydrate self-assembly, obtaining carbohydrate derivatives and using them to prepare chiral superstructures as well as precisely controlling and manipulating the morphology and function of specific superstructures for various applications remain future challenges [49,50,51,52,53,54,55,56].

Natural biopolymers have stimulated the development of synthetic analogues, while synthetic polysaccharides are still relatively unexplored in materials science and supramolecular chemistry. Synthetic polysaccharides play important roles in many biological processes, and their synthesis and application are important research directions involving multiple fields. The application of polysaccharides is very extensive. In the field of pharmacy, polysaccharides can be used for drug development, such as ingredients in tumor vaccines. The application of polysaccharides in tumor vaccines is an important research direction. By synthesizing and studying tumor-associated carbohydrate antigens, these can stimulate the body’s immune response and help fight against tumors. Considering that carbohydrates are the most abundant structural materials on Earth, the latest advances in automated synthesis and analysis technology have improved our understanding of the structure of polysaccharides, transforming our perception of polysaccharides from unformed molecules to polymers with several different conformations. This newly acquired knowledge indicates the ability to generate polysaccharides that can fold and assemble into specific structures, making it possible to design artificial polysaccharide folds and components [57].

### 2.2. Self-Assembly and Applications of Proteins

Protein self-assembly is a fundamental biological process in which proteins spontaneously organize into complex functional structures without external orientation. This process is crucial for the formation of various biological functions. However, when protein self-assembly fails, it can trigger the development of various diseases, making understanding this phenomenon extremely important. In the past decade or two, it has also been found to have great potential as an alternative pathway for manufacturing biomedical application materials [58,59,60].

Therefore, it is necessary to timely summarize the key aspects of protein self-assembly: how to use protein structure and self-assembly conditions to design biomaterials to meet different applications in the fields of biomedicine and materials. Finally, based on the cutting-edge progress made in the past few years, we have summarized the current knowledge on customizing the final function by introducing changes in self-assembly and linking it to the performance of biomaterials [61].

From a general thermodynamic perspective, the driving force of the self-assembly process is the minimization of system free energy, which is achieved by increasing intermolecular attraction and reducing intermolecular repulsive interactions. In most self-assembly processes, the formation of regular structures is determined by enthalpy, but can also be controlled by entropy, and sometimes can be controlled by both factors simultaneously [62,63,64].

**Figure 3:**
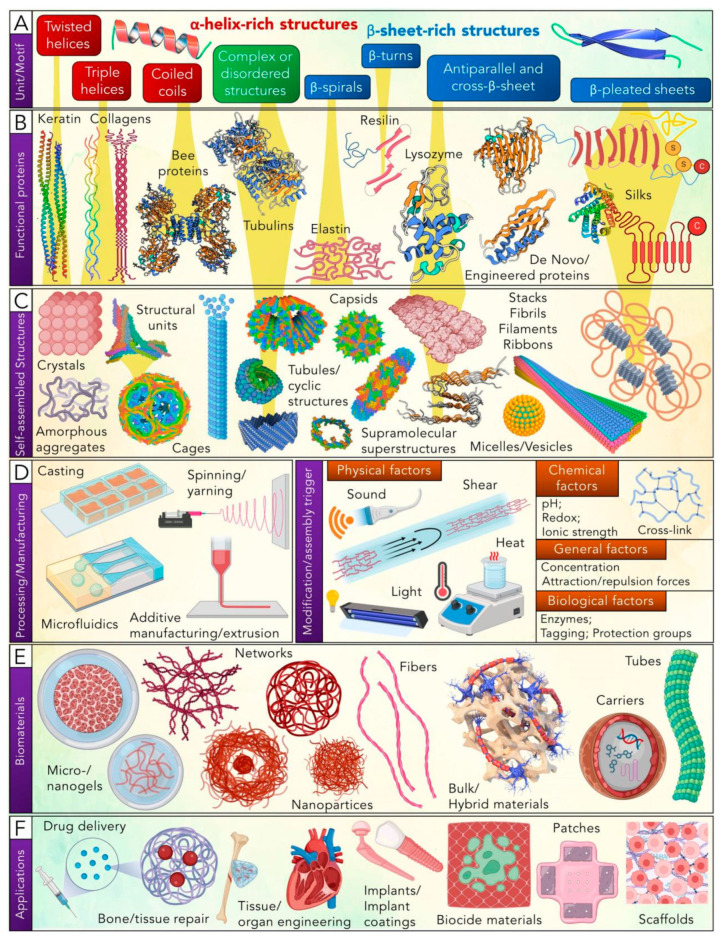
Structural hierarchy of protein self-assembly: from building blocks and structural motifs to self-assembled structures, their processing and modification, and functional biomaterials. Separate images were created with BioRender.com, Blender 2.93, and Autodesk Fusion 360 [58]. Copyright 2024. Reproduced with permission from GDCh.

According to G Whitesides and B Grzybowski’s classification, protein folding is an example of static self-assembly, which is a complex process caused by hydrophobic interactions, intramolecular hydrogen bonding, van der Waals forces, electrostatic interactions, and environmental factors. According to the structural hierarchy of protein biomaterials development (Figure 3), one of the key aspects of its design is the structure of the building blocks and precise control of protein–protein interactions due to the position of individual fragments within the protein or peptide molecules themselves [58].

Proteins can spontaneously or under the influence of so-called self-assembly triggers form various supramolecular assemblies. This is a key factor commonly used in the production of multifunctional protein biomaterials. The triggering factors for self-assembly include not only the presence of binding sites in the protein structure responsible for the process but also various environmental factors such as pH, protein concentration, ion strength, or other adjacent molecules. This also includes various physical factors such as temperature, light, [ultrasonic] sound (or pressure changes), as well as the presence of mechanical forces such as shear stress and electromagnetic force [65,66].

The other most important step in obtaining biomaterials is how to produce them. Due to their unique structures, different materials require different acquisition methods. These details include electrospinning/spinning to obtain fibers, casting/molding, as well as microfluidics and nanofluids to obtain compartments, nanoparticles, etc. In recent years, 3D printing methods based on protein-based biomaterials have shown superior performance in this field and have enabled people to manufacture many promising new materials [67].

It should be noted that differences in peptide sequences in proteins not only strongly affect the type of self-assembly but also affect the physical properties of the final biomaterials. This is particularly important for materials created for specific purposes or application areas. For example, biomaterials that require high mechanical strength or elasticity—such as those used for manufacturing implants, artificial organs, bones, or tissue regeneration—clearly must be constructed from protein assemblies where a balance between crystalline and disordered regions should be observed, and they should exhibit good enzyme stability. Proteins used as biomaterials for the manufacture of various types of membranes and gel should mainly form fibril structures. Proteins and coatings of biomaterials used for bioelectronic purposes should form stable thin films. In addition, the electrical and dielectric properties, optical transparency, thinness of the films, and chemical or thermal stability of the obtained biomaterials are also important [5,68].

#### 2.2.1. Application of Protein Self-Assembly in the Biomedical Field

##### Cell and Tissue Engineering

In the context of biomaterials, especially in the fields of tissue engineering and regenerative medicine [69], bulk materials are used to provide structural, mechanical support, and potential biological activity in biological environments. They usually constitute the main structural components of medical devices or implants [70]. In the case of biomaterials, transplantation refers to the material or tissue transplanted to a certain part of the body to repair or replace damaged tissue [71].

**Figure 4:**
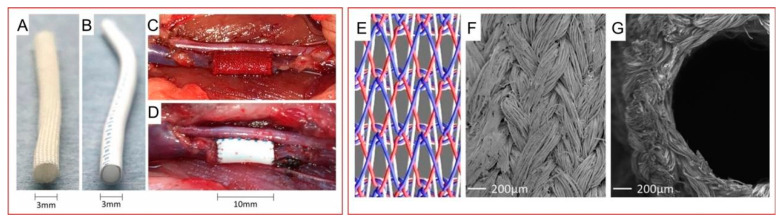
SF in comparison with the ePTFE vascular graft (**A**–**D**) and the fibrous structure of the SF graft (**E**–**G**) [73]. Copyright 2020. Reproduced with permission from Springer Nature.

Kaplan et al. reported a new set of biomaterials composed of SF based on their basic understanding of how silk fibroin, one of the most common functional biomaterials, self-assembles, including its exact composition, molecular structure, and natural spinning mechanism. These materials were obtained through heat treatment and used to directly solid-state shape regenerated silk into large “parts” or devices (rods, rods, plates, cap tubes, and screws) with adjustable mechanical properties, degradability, and swelling properties. The application of these materials as bone implants demonstrated good biocompatibility and supported the formation of new bone structures [72]. Their understanding of the self-assembly behavior of proteins, especially SF, enabled Hasegawa and his colleague to create a new vascular graft for abdominal venous system replacement. The reported patency rates for the SF group at 1 week and 4 weeks after replacement surgery were 100.0% and 94.7%, respectively. Considering the greater biocompatibility of silk, these two values were comparable to or even better than those of ePTFE grafts (Figure 4) [73].

Belluati reported artificial cells with protein-expression ability based on an enzyme-synthesized polymer. Artificial cells were synthesized through self-assembly induced by biocatalytic atom transfer radical polymerization, in which myoglobin synthesized amphiphilic block copolymers that self-assembled into structures such as micelles, worm-like micelles, polymers, and giant monolayer vesicles (GUVs). GUVs encapsulate goods during the polymerization process, including enzymes, nanoparticles, particles, plasmids, and cell lysates. The resulting artificial cells acted as microreactors for enzyme reactions and biomineralization triggered by osteoblasts. In addition, when amino acids were added, they could express proteins such as fluorescent proteins and actin. Actin aggregated in vesicles and altered the internal structure of artificial cells by creating internal compartments. Therefore, the self-assembly derived GUVs induced by biocatalytic atom transfer radical polymerization can simulate bacteria [74].

The team from the Tanden School of Engineering at New York University has developed a protein that can self-assemble into fibers and can be used for the treatment of various diseases. This is a fluorinated biomaterial composed of natural proteins that can encapsulate and deliver various drugs for treating diseases. The research team introduced a non-natural amino acid, trifluoride leucine. Due to the rarity of fluorine in the human body, biomaterials can emit light when scanned with 19FMRI (imaging using fluorine-19 nuclear magnetic resonance technology). Doctors can ensure that medication stays in the treatment area based on this. Researchers say that as a therapeutic drug, this protein can not only treat cancer or joint diseases but can also be broken down by the human body without any adverse effects. It eliminates the need for invasive surgery or biopsy, and the monitoring process is relatively easy. The research team has validated the results on a mouse model [75].

Vaccination is considered the most effective measure to prevent infectious diseases. Vaccines based on inactivated or attenuated live strains elicit immune responses by stimulating humoral and cell-mediated protective effects [76]. However, traditional vaccines are based on the production of embryonic eggs [77]. Obviously, this method is difficult to apply to potential epidemics that are rapidly erupting and is not suitable for sensitive individuals or immunocompromised individuals. We are currently researching recombinant protein-based methods as an alternative solution. Artificial protein nanoparticles provide a promising method for developing the next generation of recombinant protein vaccines [78].

Baker et al. designed various million-Dalton-level two-component icosahedral protein complexes that bind to viral receptors to manufacture multivalent nanoparticle vaccines for the prevention of HIV and respiratory syncytial virus—especially severe acute respiratory syndrome coronavirus type-2 infection—which have been further validated in several animal models [79].

In addition to preventing pathogenic infections, artificial protein assembly can further apply to cancer vaccines and immunotherapy. For example, VLP was used to carry and deliver immune adjuvants to enhance immune responses, present tumor-associated antigens to immune cells, and display specific antibodies to block immune escape. More importantly, several preclinical trials have shown promising outcomes in treatment based on VLP [80].

##### Design and Preparation of Drug Delivery Systems

Self-assembling proteins are valuable building blocks for constructing drug nanocarriers due to their self-assembly behavior, monodispersity, biocompatibility, and biodegradability [81]. Genetic and chemical modifications allow for the modular design of protein nanocarriers with effective drug encapsulation, targeting, stimulus responsiveness, and in vivo half-life. Protein nanocarriers have been developed for delivering various therapeutic molecules, including small molecules, proteins, and nucleic acids, with proven in vitro and in vivo efficacy [82,83,84].

Silk-elastin-like protein polymers (SELPs) combine the mechanical and biological properties of silk and elastin. These characteristics have led to the development of various SELP-based materials for drug delivery. Wang have recently constructed a series of SELPs (SE8Y, S2E8Y, and S4E8Y) with different silk elastic protein block ratios and described their ability to form micelle-like nanoparticles under thermal triggering. In this study, we demonstrated that doxorubicin is a hydrophobic anti-tumor drug that can effectively trigger the self-assembly of SE8Y (SELP with a silk elastic protein ratio of 1:8) into uniform micelle-like nanoparticles. This drug can be loaded into SE8Y nanoparticles. This study provides a simple method for preparing nanoscale drug-loaded SELP packages with potential for tumor cell therapy [85].

Li designed and synthesized a nine peptide (NapFFKKFKLKL), which can be co-assembled with dexamethasone sodium phosphate (Dexp) to produce NapFFKKFKKLKL/Dexp supramolecular hydrogel for eye administration. Using the rat model of experimental autoimmune uveitis (EAU), they proved that the obtained NapFFKKFKLKL/Dexp hydrogel has an effective ability to reduce intraocular inflammation and maintain the morphology of the retinal structure. In conclusion, the obtained NapFFKKFKLKL/Dexp hydrogel may be a promising drug carrier system [86].

Transferrin is also used to treat autoimmune diseases such as myasthenia gravis (MG). In MG patients, autoantibodies recognize and block the nicotinic acetylcholine receptor (AChR). By fusing the alpha subunit of AChR with transferrin, Keefe et al. created SHG2210, a fusion protein that can be recognized and bound by anti-AChR autoantibodies and internalized by transferrin receptors [87].

Like transferrin, ferritin is also used for targeting and carrying drugs. Ferritin is an iron storage protein composed of 24 subunits, assembled into spherical nanocages [88]. Although this nanocage naturally accommodates up to 4500 iron atoms, it can also be designed to accommodate various therapeutic drugs [89]. Its loading capacity depends on several factors, including the molecular weight of the drug, encapsulation strategy, and the selection of co-solvents for embedding the drug [90].

Jiang et al. synthesized cobalt nanoenzymes in ferritin nanocages, which were nanomaterials with intrinsic enzyme-like activity. Compared with nanoenzymes synthesized outside the nanocage, synthesizing nanoenzymes within the nanocage provided better control over the size and structure of the nanoenzymes. The method employed used recombinant and fused liver-cancer-targeting peptides with ferritin to display them outside the nanocage for optimal targeting. Although this design was used for diagnostic purposes, the significant targeting specificity it achieves indicated that it can be easily used as a drug delivery carrier [91].

These studies emphasized the multifunctionality and versatility of protein-based drug delivery materials. These carriers had excellent biocompatibility, biodegradability, and minimal toxicity. The continuous progress in genetic engineering and synthetic biology has enabled this work, making it possible to combine new chemical substances and structural motifs into peptide sequences to improve drug coupling, targeting, stimulus responsiveness, and self-assembly [92,93,94].

Li proposed a universal self-assembly strategy to construct multi-enzyme nanostructures based on synthetic protein scaffolds, utilizing the spontaneous protein reaction between SpyCatcher and SpyTag to form a protein scaffold. Two types of protein scaffolds were generated: two types of skeletal proteins were crosslinked and graded to form multiphase nanostructures (cross-linked scaffolds), and sequential enzymes from the biosynthesis pathway of menaquinone were assembled on both scaffolds. Both of these scaffold components effectively increased the yield of the final product of the intermediate catalytic reaction in the biosynthesis of menaquinone. In summary, by combining the SpyCatcher/SpyTag reaction and docking domain interactions, the self-assembly of sequence enzymes produced unique structures, catalytic activities, and unexpected catalytic mechanisms [95].

#### 2.2.2. Applications of Protein Self-Assembly in the Field of Materials

Yang et al. showed that natural LecA proteins can self-assemble into all types of structures: from one-dimensional nanobelts and nanowires to two-dimensional nanosheets and three-dimensional layered structures. In addition, self-assembly can be easily controlled by controlling the length of ligands, thereby inducing the assembly of protein molecules. By changing the number of repeating units in the ligand, the size of the assembly can be controlled without the need for protein engineering. When the assembly type in bulk materials can be controlled, this is particularly important in creating biomaterials with the required performance [96].

Duraj Chatte started to develop a kind of biological ink, called “microbial ink”, which is completely produced by genetically engineered microbial cells. The program was designed to assemble protein monomers into nanofibers from bottom to top, further forming a nanofiber network including extrudable hydrogels. By embedding programmed *Escherichia coli* (*E. coli*) cells and nanofibers into microbial ink, the 3D printing of functional live materials was further demonstrated. Microbial ink can isolate toxic parts, release biological products, and regulate its own cell growth through chemical induction of a well-designed genetic circuit. In this work, the advanced capabilities of nanobiotechnology and biomaterials technology in the 3D printing of functional living buildings were demonstrated [97].

Peydayesh converted natural proteins into amyloid fibrils and demonstrated that the aggregation of cationic protein amyloid proteins and anionic linear polysaccharides led to interfacial self-assembly of biomaterials, and precisely controlled their structure and properties. These results indicated that agglomerates were a primitive and effective biomaterial with multiple uses in internal medicine [98].

Shapiro designed high electronic conductivity in the pili produced by a genome-encoded *E. coli* strain. Adding tryptophan to the pili increased the conductivity of individual filaments by more than 80 times. This work demonstrates the potential for manufacturing advanced conductive protein nanowires and hybrid biomaterials [99].

**Figure 5:**
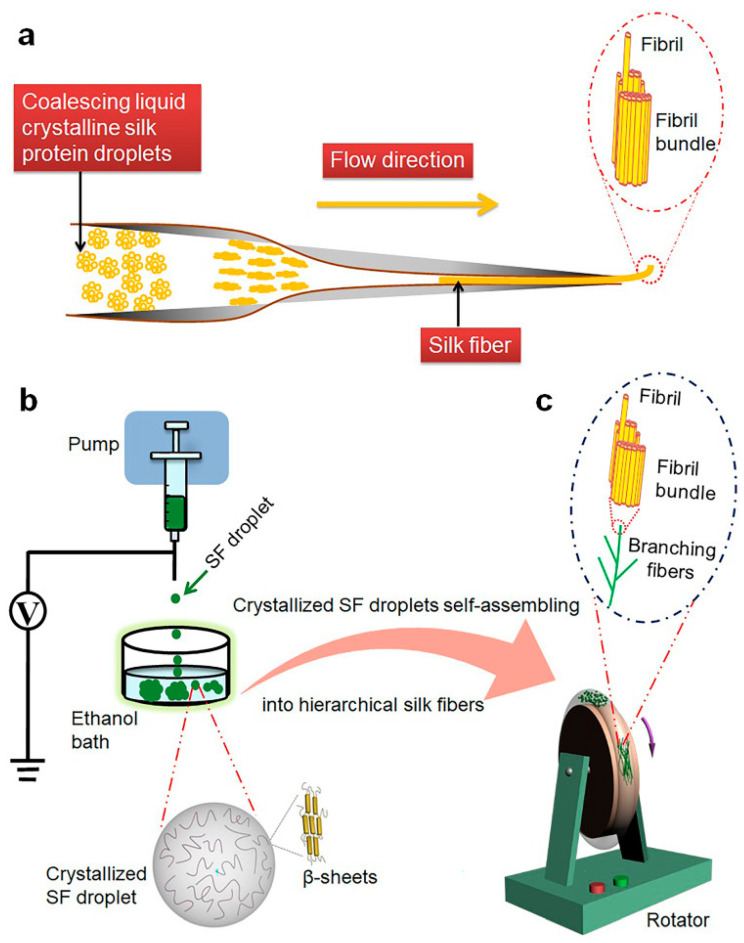
(**a**) Schematic mechanism illustration of the liquid crystalline model for formation of in vivo silk fibers, which was drawn based on [10,11,15]. (**b**) Formation of crystallized droplets from silk fibroin (SF) molecules. (**c**) Crystallized SF droplets self-assembling into hierarchical suprafibrillar silk fibers [100]. Copyright 2021. Reproduced with permission from Nature Publishing Group.

Inspired by spider liquid crystal spinning, Fan reported that nanofiber bundles were arranged along the long axis through the self-assembly of crystalline silk fibroin (SF) droplets. The formation of self-assembled SF fibers is a process of coalescing droplets and sprouting to form a branching fiber network, similar to the development of capillaries in our body (Figure 5). The assembled graded SF fibers had high biological activity and can significantly enhance the diffusion and growth of human venous endothelial cells compared to natural SF fibers. This work helped to understand the natural silk production process of spiders and provides strategies for designing and developing advanced fiber biomaterials for various applications [100].

Biological evolution has produced precise and dynamic nanostructures that can respond to pH and other environmental conditions for recombination. For example, spider silk protein controls the generation of solid silk through pH-sensitive relays, and CTP synthase aggregates under low pH conditions to help yeast cells maintain homeostasis during starvation. These natural phenomena have sparked the interest of bioengineers in designing pH-dependent protein materials, which have potential applications in fields such as tissue engineering and self-healing biomaterials [101].

Inspired by the self-assembly ability of the stratum corneum proteins of the Asian corn borer caterpillar *Ostrinia furnacalis*, Li spontaneously formed nanocapsules of insect stratum corneum peptides through a one-step solvent exchange method. The concentration gradient generated by the mixture of water and acetone promoted the localization of peptides and self-assembly into hollow nanocapsules. Research found that the intrinsic affinity of peptides to specific solvent concentrations was the fundamental driving force, while the diffusion of water and acetone formed a gradient interface, triggering peptide localization and self-assembly. This gradient-mediated self-assembly provided a revolutionary approach for the simple generation of peptide-based nanocapsules [102].

The team led by Professor Georg K. A. Hochberg and Professor Jan M. Schuller from Germany reported the discovery of a natural protein, citrate synthase, which derives from the cyanobacterium *Chlorella longum*, and found that this enzyme can self-assemble into the Sierpiński triangle [103].

Undoubtedly, the number of new protein-based materials is increasing proportionally every year, sometimes even faster than our understanding of the strategies to obtain them and the ability to describe their characteristics. In addition, there are still many things that are not within the scope of this current work but are equally interesting and need to be discussed separately. Considering the current trends in machine learning development and accumulated knowledge, this algorithm can now be used to predict the properties of protein-based supramolecular assemblies, which can be considered a promising candidate for designing biomaterials in the future. With the development of new materials, strengthening biological and material science research can improve tools for predicting their behavior and improving their design. For example, experimental results will promote theoretical models and molecular simulations, thereby enhancing the de novo design of protein structures. This will help us understand the structure–function relationship so that we can design new forms to control the delivery of therapeutic agents. It is also necessary to continue developing tools to improve the production of protein materials. The current methods suffer from low yield, limited selection of post-translational modifications, or high levels of endotoxins. Researchers can address these challenges by exploring alternative systems including bacteria, yeast, mammalian, and plant cells [104,105,106].

The follow-up progress of biomacromolecule assemblies should focus on integrating modified and natural functions to achieve complex cooperation among various abilities and multifunctional unity. Therefore, the design of self-assembled biomaterials for clinical use in the future requires communication, integration, and collaboration with the living system, which exhibits different functions based on the external environment. Compared to synthetic building blocks, natural building blocks are extremely complex and have different assembly paths, generating multiple structures to support life processes. As a necessary foundation, using complex biomolecules to construct more complex structures is crucial for supporting multifunctionality and intelligence. Additionally, another noteworthy thing is polymorphism, which uses the same building blocks to build different assemblies. By adjusting thermodynamic or kinetic processes alone, different assembly pathways can be controlled to produce products close to life systems, thereby generating and transforming polymorphic assemblies. The self-assembly of biomolecules as a new research field is still in its early stages. Although there are many areas that require attention, new methods and ideas have become prominent in solving very challenging problems in medicine and industry through interdisciplinary integration. For example, combining synthetic biology techniques to directly produce biomolecular components inside or outside the organism can produce underwater adhesives, light-regulated biofilms, biosensors, and so on [107,108,109]. With a deeper understanding and design of biomacromolecules, we believe that the dream of fully utilizing biomacromolecule materials in medicine and industry will become a reality.

## 3. Self-Assembly and Applications of Non-Natural Polymers

There are various types of non-natural polymers with clear structures that are designed into materials with different structures and applications through self-assembly, and their applications are also more extensive. While focusing on technology and applications, it is often necessary to consider more environmental compatibility, safety, and practical value. Polymerization-induced self-assembly (PISA) is a newly developed polymer self-assembly technology from the past decade, which involves simultaneously achieving polymerization and self-assembly processes in the same system. In a typical PISA process, macromolecular initiators (also used as stabilizers) are first dissolved in the solvent system; then, with the polymerization of monomers, the generated second block gradually becomes longer and its solubility gradually decreases, driving the in situ self-assembly of block copolymers to form micelles; furthermore, monomers continue to polymerize in micelles, which evolve into nano self-assembled bodies with various morphologies such as spherical, worm-like, vesicle-like, etc. Therefore, PISA technology can achieve the self-assembly of polymers with high solid content (up to 50 wt%) through a “one pot” process [110,111]. In this section of the review, important applications of other self-assembly techniques are also included, which will provide valuable references for future research in related fields.

### 3.1. Applications in the Field of Biomedicine

Professor Robert J. Hickey proposed a new strategy based on block polymer self-assembly to prepare high-performance actuators by simulating the stripe structure of skeletal muscle fibers. By combining solution-phase self-assembly of block copolymers and strain-programmed crystallization (SPC), flexible actuators/artificial muscle fibers with excellent mechanical and actuation performance and recyclability can be manufactured [112].

Aliabadi synthesized three different sizes of PLA-b-PAPMA (polylactic acid-b-poly(N-(3-aminopropyl) methacrylamide)) diblock copolymers for the first time to prepare polymer micelles for gene delivery. In this copolymer, the p (APMA) block with hydrophilic cationic properties can serve as a nucleic acid condensation platform and a shell of micelle structure during self-assembly. It can be concluded that cationic micelles with a molecular weight ratio of hydrophilic segments to the total mass of the polymer of about 60% and greater than 50% have higher stability, condensation ability, and transfection efficiency, which may be attributed to better self-assembly processes and better exposure of amino groups on the surface of the micelles [113].

Yao proposed the ring-opening polymerization-induced self-assembly (ROPISA) of poly (ethylene glycol)-poly (salicylic acid) block copolymers in tetrahydrofuran (THF) as a higher-order nanostructure. ROPISA involves rapid ring-opening polymerization-induced self-assembly of salicylic acid O-carboxylic anhydride (SAOCA) within a few minutes, followed by slow in situ self-assembly, leading to a non-equilibrium assembly process. This dynamic driven component can achieve the worm-like structure required for biomedical applications [114].

Self-classification plays a crucial role in the biological system, such as the selective assembly of DNA and the specific folding of proteins; however, the self-selection of artificial spiral polymers is rarely achieved. In this work, a single-handed helical poly (phenylisocyanide) probe with pyrene (Py) and naphthalene (Np) was prepared, which showed interesting self-sorting properties in solution, solid, gel, and on gel surface, driven by helicity and molecular weight (Mn). Polymers with the same helicity and similar molecular weight can self-classify and assemble into a clear two-dimensional smectic structure, and can form a stable gel in organic solvents. On the contrary, mixed polymers with opposite rotation or different molecular weights repel each other and do not aggregate. In addition, gel with helical polymers with the same chirality and similar molecular weight can recognize themselves and stick together to form the gel [115].

Although the self-assembly technology of non-natural polymers has matured, its application research is still in the exploratory and preliminary stage, and further exploration and development are needed. Some problems encountered in its practical application process, such as the stability and purification of nano self-assembled bodies and the possible toxicity of residues, also need to be solved. These should be the focus of future research on non-natural polymer self-assembly. Overall, the in-depth development of non-natural polymer self-assembly technology can bring new opportunities for the development of various fields [116].

### 3.2. Applications in the Field of Materials

#### 3.2.1. Self-Assembly and Application of Amorphous Structured Materials

Through the method of using hydroxyl-terminated polyisobutylene (PIB-OH), diphenyl phosphate (DPP), cyclohexane, ε-caprolactone (ε-CL), and/or δ-pentolactone (δ-VL) as the stabilizers/macromolecular initiators, catalysts, solvents, and monomers, ring-opening polymerization-induced self-assembly (ROPISA) was achieved. ROPISA has introduced biodegradable, biocompatible, and crystallizable poly (lactone) into nanoparticles and related materials. Temperature regulation altered the monomer sequence in the P (CL-co-VL) block, as well as subsequent crystallization and self-assembly. It was interesting that at different polymerization temperatures, even if the composition was similar, nanoparticles exhibited different forms. The results of this study indicated that ROPISA provided a feasible alternative for synthesizing nanoparticles in PISA [117].

**Figure 6:**
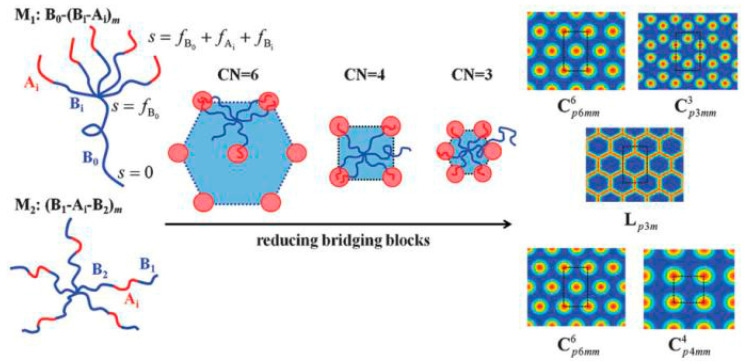
(**Left**): Schematic plot of the architectures of the two designed AB-type multiblock copolymers, M_1_ and M_2_. A and B blocks are drawn in red and blue colors, respectively. Center: illustrative plot of chain configurations in three typical cylindrical morphologies with different coordination numbers demonstrating the formation of bridges among neighboring domains and their impact on the domain arrangement (i.e., packing lattice). (**Right**): density profiles of the stable morphologies self-assembled from the two block copolymers, including the classical phase of hexagonal cylinders. Copyright from American Physical Society [118]. Copyright 2016. Reproduced with permission from APS.

Inspired by the previous research on self-assembly of ternary block copolymers, Professor Qiu Feng’s research group led by Professor Li Weihua and others designed the topological structure of AB-type block copolymers and introduced bridging units into multi-arm polymers. For the first time, the concept of “combinatorial entropy” was used to propose an entropy-driven mechanism for regulating the self-assembly of block copolymers. The self-consistent field theory calculation of the system verified this mechanism and successfully predicted the non-traditional phase structure of the tetragonal arrangement columnar equality (Figure 6). This study broadens the regulatory approach for the self-assembly of block copolymers; meanwhile, the exploration of the conditions for the formation of tetragonal structures in AB-type block copolymers also provided theoretical guidance for related experimental research and industrial applications [118].

Liquid–liquid phase separation (LLPS) of biopolymers has recently been shown to play a central role in the formation of membraneless organelles with multiple biological functions. The continuously growing fibers obtained through supramolecular polymerization of synthetic components are responsible for separating the phase into highly anisotropic aqueous droplets (tactoids) through an entropy-driven pathway. The crowded environment regulated by the concentration of dextran not only affects the kinetics of supramolecular polymerization but also affects the properties of LLPS, including the phase separation kinetics, morphology, internal ordering, fluidity, and mechanical properties of the final corn kernels. In addition, the substrate–liquid and liquid–liquid interfaces have been shown to accelerate the LLPS of supramolecular polymers, allowing for the generation of numerous three-dimensional ordered structures, including highly ordered arrays of surface micrometer-long triangles. Several supramolecular polymers have demonstrated universality and the many possibilities of supramolecular polymerization in controlling emerging forms, opening up a new field of matter, from highly structured aqueous solutions stabilized by LLPS to nanoscale soft materials [119].

**Figure 7:**
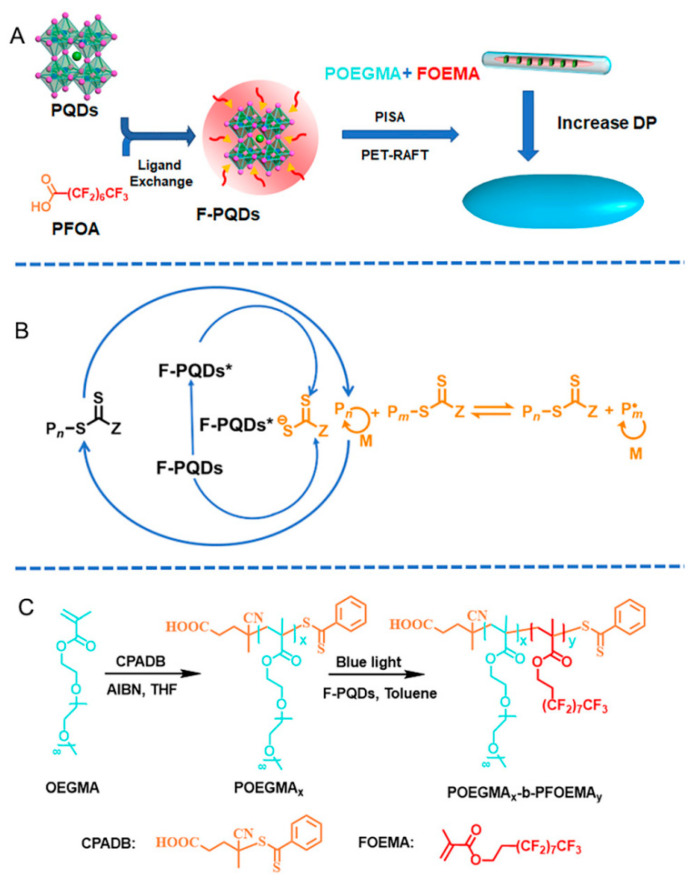
(**A**) Schematic demonstration of the surface-ligand-exchange strategy of PQDs and the PISA process based on PET-RAFT polymerization, (**B**) the proposed PET-RAFT polymerization mechanism of F-PQDs, and (**C**) a synthetic approach for producing the diblock copolymer POEGMA-b-PFOEMA [120]. Copyright 2024. Reproduced with permission from the Royal Society of Chemistry.

Perfluorooctanoic-acid-modified CsPbBr3 perovskite quantum dots (F-PQDs) were used as luminescent centers and photocatalysts to prepare organic–inorganic hybrid nanocomponents. The polymerization-induced self-assembly (PISA) technique of poly (ethylene glycol) methyl methacrylate monomethyl ether)-b-poly (perfluorooctyl) ethyl methacrylate copolymer (POEGMA-b-PFOEMA) using photo-induced electron/energy transfer RAFT (PET-RAFT) simplifies the synthesis steps of hybrid nanoparticles and enables in situ encapsulation of PQDs through dipole–dipole interactions based on F-PQDs surface fluorocarbon chains and FOEMA. The insolubility of PFOEMA blocks with liquid crystal properties allowed for effective modulation of the hybrid nanostructures in toluene. The adjustment of the block lengths achieved a transition from nanorods to spindle-shaped nanocomponents, and these mixed nanoparticles possessed inherent fluorescence and enhanced stability of the PQD (Figure 7). This strategy simplified the preparation scheme of PQD/polymer composite materials and provided a new perspective for the design of organic–inorganic hybrid materials through the optical PISA strategy [120].

Liu quantitatively studied the additivity of effective two-body interactions between spherical nanoparticles in layered structures formed by block copolymers using self-consistent field theory. The self-assembly behavior of nanoparticles with these non-additive attractive interactions has been well addressed. By comparing the conformational characteristics of polymers in systems with different nanoparticle aggregation structures, it was revealed that the relative size of polymers and nanoparticles can be used to regulate the competition between the depletion effect and polymer conformational entropy, thereby regulating the entropy-driven mechanism of directed self-assembly of nanoparticles in block copolymer (BCP) behavior. This new mechanism can be applied to the directed assembly of nanoparticles in various periodic structures formed by block copolymers, and can also be used for the design of novel self-assembly structures for isotropic nanoparticles [121].

Shao demonstrated the use of ultrasound (20 kHz)-assisted ethanol phase RAFT-PISA to prepare nano components with controllable morphology and size within three hours. Using N, N-dimethylaminoethyl methacrylate as a macromolecular reversible addition–fracture chain-transfer agent (PDMA-CTA) to control the nucleation monomer benzyl methacrylate (BzMA), nano assemblies with different morphologies were obtained [122].

The metal-free organic photocatalysts used for photo-mediated–reversible-deactivation radical polymerization (photo-RDRP) have made increasing progress in the precise synthesis of polymers. However, there are still challenges in developing efficient and environmentally sustainable carbon dots (CDs)-based organic catalysts. Xiao prepared N-doped CDs derived from phenanthroline derivatives (Aphen) as non-metallic photocatalysts for photo-induced electron transfer–reversible-addition broken-chain transfer (PET-RAFT) polymerization. The introduction of the phenanthroline structure has successfully demonstrated for the first time the construction of fluorescent polymer nanoparticles using photo-induced and polymerization-induced self-assembly (Photo-PISA) technology, using CDs as photocatalysts and phosphors [123].

Polymer nanotubes with hollow and anisotropic structures have aroused great interest in materials science. However, according to the theoretical prediction of stacking parameters, it is difficult to generate polymer nanotubes during the self-assembly of block copolymers, which may be due to the lack of directionality in hydrophobic solvent interactions. Here, Zhu demonstrated that the aromatic interactions between hydrophobic solvent blocks promoted the formation of polymer nanotubes. Aromatic monomer 2- (methacryloxy) ethylanthracene-9-carboxylic acid ethyl ester (MAEAC) was subjected to reversible-addition chain transfer (RAFT) dispersion polymerization using polyethylene glycol as a macromolecular RAFT agent (PEG45-CPADB), resulting in polymer nanotubes of significant length (>11 μm) [124].

#### 3.2.2. Self-Assembly and Application of Forming Ordered Crystal Structures

Ning Yin demonstrated a universal approach to regulate the spatial distribution of guest species within host inorganic crystals through doping strategies. A well-defined block copolymer nanoparticle, poly (methacrylic acid) x block poly (styrene alt-N-phenylmaleimide) y (PMAx-P (St alt NMI) y), was synthesized through polymerization-induced self-assembly. These anionic nanoparticles can be self-assembled onto the surface of larger cationic nanoparticles through electrostatic interactions, forming colloidal nanocomposite particles (CNPs) [125].

Mixed conductors—materials that can effectively conduct ions and electrons—are an important class of functional solids. Christopher J. Takacs from the SLAC National Accelerator Laboratory, Alberto Salleo from Stanford University, and Alexander Giovannitti demonstrated an organic nanocomposite material that spontaneously formed when organic semiconductors were mixed with ionic liquids and exhibited effective room-temperature mixing conduction. They used a known polymer that formed a semi-crystalline microstructure to insert ions into the side chain domains of the crystal, thereby maintaining the integrity of the electron transport pathway. Therefore, the obtained material was ordered, manifested as the alternation of rigid semiconductor chips and soft ion conductive layers. This unique dual network microstructure led to a dynamic ion/electron nanocomposite material with liquid-like ion transport and highly mobile electronic charges [126].

Hueckel prepared ionic colloidal crystals in water using a method called polymer decay Coulomb self-assembly. The key to crystallization was to use neutral polymers to maintain clear distances between particles, allowing us to adjust the attraction overlap of the double layer and guide particles to disperse, crystallize, or permanently fix as needed. By using the Debye shielding length to fine-tune the assembly, the nucleation and growth of macroscopic single crystals has been demonstrated with various colloidal particles and commercial polymers, selecting ion colloidal crystals that were isomorphic to cesium chloride, sodium chloride, aluminum diboride, and K4C60 based on the particle size ratio. Once fixed by simply diluting the solution salt, the crystal can be extracted from the water for further operation, demonstrating the accurate transformation from the solution phase assembly to a dry solid structure [127].

Researchers have designed and constructed a self-assembled planar structure that formed crystalline polymers through nucleation growth, utilizing surface charges to enhance the stability of the self-assembled planar structure. Ultrasound fragmentation was used to obtain copolymer micelles with crystal nuclei as seed crystals, and homopolymers with terminal charges were added to the copolymer micelles to prepare uniformly sized and controllable block copolymers. The process of seed-induced growth can be characterized by two-dimensional crystal morphology based on the shape memory effect of hexagonal or rectangular precursor crystals. This novel method was described through two different polymer systems and provided a new direction for the creation and widespread application of two-dimensional multilayer structures [128].

Silicon dioxide microspheres (SM) must have ideal monodispersity, narrow particle size distribution, and high sphericity in order to prepare photonic crystals (PC) and other materials, such as microsphere reference materials. Chen X adopted techniques to increase the concentration of reactants and increased the temperature to improve the synthesis rate of SM. SM with uniform particle size (polydispersity index less than 0.05) and good spherical characteristics was prepared through uniform nucleation. Through self-assembly of SM, highly ordered PC with dense stacked opal structure was obtained (stacking faults of about 1.5% and point defects of about 10^−3^). The PC with inverted opal structure were used to test their response characteristics to ethanol, demonstrating good performance. This research may provide important insights for the development of other types of microspheres [129].

**Figure 8:**
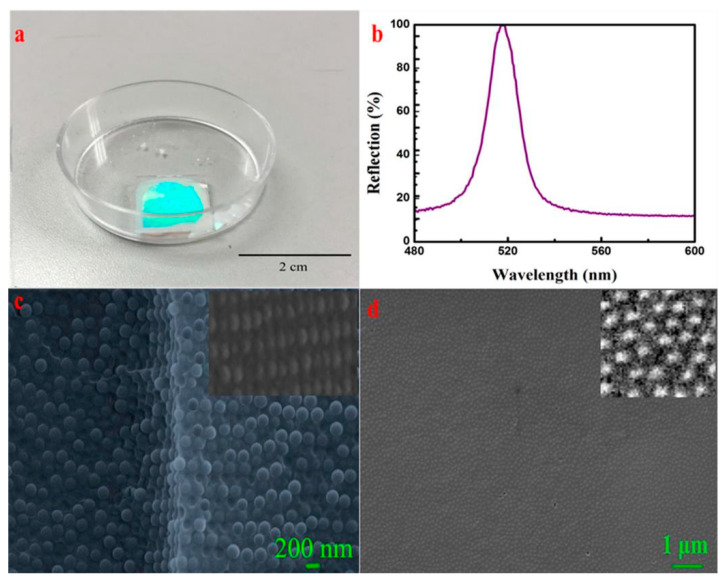
Ordered CC structure appeared on the planar interface using the fixed proportion of soft matter system. (**a**) Optical photograph of the CCs, (**b**) reflection spectrum of the CCs, (**c**) SEM image of the cross-section of the CCs, (**d**) surface SEM image of the CCs [130]. Copyright 2019. Reproduced with permission from MDPI.

Cracks and defects may result in lower reflectivity and larger full width at half maximum (FWHM), which are the main obstacles to obtaining highly ordered structures of colloidal crystals (CC). A high-quality CC with high reflectivity (over 90%) and a 9.2 nm narrow FWHM was successfully prepared using a fixed ratio of a soft system composed of silica particles (SP), polyethylene glycol diacrylate (PEGDA), and ethanol, clarifying the effects of the refractive index difference, volume fraction, and particle size on FWHM. Firstly, the influence of planar and curved interfaces on self-assembly was elucidated. CC was successfully prepared on a planar interface (Figure 8) but unfavorable results were observed on a curved interface. Secondly, a hard sphere system composed of SP, PEGDA, and ethanol was established, and the entropy-driven phase transition mechanism of the polydisperse system was elucidated [130].

## 4. Discussion, Conclusions, and Future Directions

### 4.1. Summary of Research Progress on Polymer Self-Assembled Materials

The research field of polymer assembly materials has been rapidly developing, and the following outlines the recent progress. Multi-level self-assembly structure: Researchers have achieved the construction of multi-level self-assembly structures by combining different types of nanostructure units. This method expands the functionality and complexity of self-assembled nanomaterials, providing a new approach for preparing nanodevices with specific properties [131]. Bioinspired self-assembly: Inspired by the molecular tissues within living organisms, researchers have conducted extensive research on self-assembled nanomaterials. By mimicking biological processes such as protein folding and cell self-assembly, nanostructures with biocompatibility and functionality have been successfully constructed, such as biomimetic bones, artificial cells, and drug delivery systems [132]. Attributed control of self-assembled nanoparticles: By adjusting the experimental conditions and material selection during the self-assembly process, researchers can control the morphology, size, crystal structure, and surface properties of nanoparticles. This property control can not only optimize the performance of nanomaterials but also achieve precise control of their optical, magnetic, and electrical properties [133]. The application of self-assembled nanomaterials in energy storage and conversion: Self-assembled nanomaterials have enormous potential in the energy field. Researchers have successfully applied self-assembled nanomaterials to energy storage and conversion devices such as lithium–ion batteries, supercapacitors, photocatalysts, and fuel cells, improving the performance and stability of energy devices [134]. The application of computational simulation and machine learning: In recent years, computational simulation and machine learning methods have been increasingly applied in the research of self-assembled nanomaterials. By simulating and predicting the self-assembly process and the relationship between its structure and properties, the speed of material design and optimization has been accelerated, providing guidance for experiments.

These research advances provide strong support for the application and theoretical development of self-assembled nanomaterials and also open up new perspectives for the development of more complex and functionally diverse self-assembled nanomaterials. In the future, with the emergence of more cutting-edge technologies, self-assembled nanomaterials will continue to demonstrate enormous potential and application prospects in many fields.

### 4.2. The Advantages of Self-Assembled Materials

Self-assembly ability: Self-assembly is a spontaneous process in which nanoparticles or molecules can arrange and assemble themselves into stable structures based on mutual forces. This ability makes it easier to prepare complex nanostructures without the need for complex equipment and expensive costs. Controllability: The self-assembly process can precisely control the morphology, size, and structure of nanomaterials by adjusting experimental conditions and material properties. This controllability enables the performance of nanomaterials to be designed and optimized on demand, meeting the needs of different application fields. Multifunctionality: Self-assembling nanomaterials can achieve a combination of multiple functions. By selecting different types of nanounits or regulating self-assembly processes, various functions such as electronics, optics, magnetism, and biological activity can be integrated into nanomaterials. Structural complexity: Self-assembling nanomaterials can construct highly ordered and complex structures, such as nanocrystals, nanochannels, and layered structures. These structures have unique properties and functions and can be applied in fields such as nanoelectronics, sensors, catalysts, etc. Energy and cost-saving: Compared to traditional preparation methods, self-assembled nanomaterials typically require less energy and resources. It can be carried out at room temperature and atmospheric pressure without the need for expensive equipment and specialized laboratory environments, thereby reducing the energy consumption and cost of the preparation process. Scalability and productivity: The preparation method of self-assembled nanomaterials can be used for large-scale production, suitable for industrial production needs. Meanwhile, due to the high efficiency and controllability of self-assembly, material performance consistency and stability can be achieved, providing the possibility for commercial applications [135].

### 4.3. Future Development

Nowadays, carbohydrate, protein, and peptide macromolecular biomaterials have been used in all aspects of medicine, including synthetic scaffolds for in vitro organ culture, hydrogels for wound healing, and macromolecular implants for bone repair. Standing on the shoulders of giants, the future demands precision and personalized medicine, seeking the next generation of biomaterials with “intelligent” functional characteristics, including accurate identification, self-organization, adaptability, and self-regulation. This requires biomaterials to not only coexist but also integrate with living systems. To manufacture advanced biomaterials, it is natural to consider how living systems can utilize biomaterials to achieve complex, ordered, and accurate behavior. Precision biomacromolecule assemblies have many advantages, such as nanoscale precision, multi-dimensional controllable microstructures, endogenous production, easy integration with living cells, dynamic and hierarchical characteristics, and inherent biological activity for transmitting biological signals. These advantages may be difficult to achieve with existing biomaterials. Although various artificial nucleocapsids have been manufactured using protein building blocks in the laboratory, none of them have the same multi-component synergistic effect as natural viral particles. Therefore, the precise self-assembly of carbohydrates and proteins as emerging biomaterials for future medical needs remains an untapped virgin field, as their self-assembly behavior is more difficult to predict, prepare, and design.

The polymer nano-assemblies prepared by synthesizing polymer self-assembly have potential applications in the field of drug delivery. In the process of preparing drug delivery nanocarriers, two aspects usually need to be considered: drug encapsulation and drug release. By utilizing the self-assembly function of block polymers, block polymers are also commonly used in the preparation of polymer films. By selecting parameters such as the composition, solvent, and concentration of block copolymers, the pore structure and size of the membrane can be controlled, and ultimately the separation selectivity of the membrane can be regulated. Similarly, the nano self-assembled materials obtained during the PISA process can also be used for membrane preparation and application. Hydrogel is a three-dimensional hydrophilic network structure formed by polymers. Hydrogels with environmental responsiveness can also be stimulated externally, showing controllable intelligent behavior, which has attracted extensive attention of researchers. Using nano self-assembly as a cross-linking point provides a new idea and application for the preparation of hydrogels. Overall, with the unremitting efforts of researchers, the self-assembly system of synthetic polymers has become increasingly perfect. The controllable morphology of self-assembled bodies and the advantages of high solid content preparation enable PISA technology to be better extended to various application fields. Although the self-assembly technology of synthetic polymers has become relatively mature, its application in research is still in the exploratory and preliminary stage, and further exploration and development are needed. Some problems encountered in practical applications, such as the stability and purification of nano self-assembled bodies as well as the possible toxicity of residues, also need to be further solved. These should be the focus of future research on synthetic polymer self-assembly technology.

The future development of polymer self-assembled materials will continue to promote the progress and application of nanotechnology. Here are some possible future development trends. Multifunctionality and intelligence: In the future, self-assembled nanomaterials will place greater emphasis on multifunctional and intelligent design. By combining different types of nano units and functional units, multiple functional integrations of nanostructures can be achieved, such as optics, electronics, magnetism, and biosensing. Meanwhile, utilizing intelligent design and responsiveness, nanomaterials can automatically adjust their structure and performance based on external stimuli or environmental changes. Accurate control and scalable preparation: With the continuous development of nanomaterial preparation technology, the precise control and scalability of self-assembly processes will be improved. Through more precise experimental conditions and theoretical simulations, more precise control of the structure and properties of nanomaterials can be achieved. Meanwhile, developing efficient and low-cost preparation methods, such as solution impregnation, printing, spraying, etc., can achieve large-scale production and application. Self-repair and sustainability: Future self-assembled nanomaterials may have the ability to self-repair, automatically repairing their own structure and function after damage. In addition, researchers will also strive to develop renewable and degradable materials to reduce their impact on the environment in response to the requirements of environmental friendliness and sustainable development. The combination of biomolecules and synthetic polymers provides a simple way to leverage the advantages of the synthetic world and nature. This is not only important for the development of new innovative materials but also promotes the application of biomolecules in various fields such as medicine, catalysis, and water treatment. Due to the rapid progress in polymer nanomaterial synthesis strategies and a deeper understanding of the structure and function of biomolecules, the construction of advanced polymer-based bio-hybrid nanostructures (PBBNs) has become promising and feasible [136,137]. Nanodevices and applications: Self-assembling nanomaterials have broad potential in nanodevices and applications. For example, self-assembled nanoparticles can be used to prepare efficient solar cells, photoelectric sensors, high-density storage media, and topological quantum computing. In addition, self-assembled nanomaterials can also be applied in fields such as drug delivery, biological imaging, nanorobots, and flexible electronics. Biomimetic and biomedical applications: Inspired by molecular tissues within living organisms, research on self-assembled nanomaterials in the fields of biomimetic and biomedical applications will also be promoted. By imitating the self-assembly process of biological systems, more precise and intelligent biomimetic materials can be constructed and applied in tissue engineering, drug delivery, disease diagnosis and treatment, and other fields [138].

In summary, future development will further promote the application of self-assembled nanomaterials in various fields and encourage innovation and development of nanotechnology. With the continuous progress of technology and the deepening of research, we can expect to see more self-assembling nanomaterials with multifunctionality, intelligence, and sustainability emerge.
